# Cutaneous Cysticercosis Mimicking an Epidermal Inclusion Cyst: A Rare Case

**DOI:** 10.7759/cureus.84328

**Published:** 2025-05-18

**Authors:** Sunidhi Rajput, V Ramalakshmi, Anjali Rajpoot

**Affiliations:** 1 General Surgery, Sree Balaji Medical College and Hospital, Chennai, IND; 2 Department of Biology, Banasthali Vidyapith, Jaipur, IND

**Keywords:** cutaneous cysticercosis, cysticercosis, epidermal inclusion cyst, histopathology, taenia solium, ultrasonography

## Abstract

Cutaneous cysticercosis is a rare clinical manifestation of the larval stage of *Taenia solium* infection. While neurocysticercosis is the most commonly reported form, subcutaneous presentations are often overlooked or misdiagnosed due to their asymptomatic, benign, and cyst-like appearance. The parasite’s ability to mimic common dermatologic lesions, including lipomas or epidermal inclusion cysts, complicates timely diagnosis. This underscores the importance of thorough evaluation, especially in endemic regions, and highlights the utility of histopathological and imaging studies. A 23-year-old immunocompetent male presented with a two-week history of a painless, cystic swelling over the right subcostal region, without signs of inflammation or discharge. Ultrasound of the superficial soft tissue revealed a well-circumscribed cystic lesion suggestive of an epidermal inclusion cyst. Surgical excision of the 0.5 × 1 cm bean-shaped swelling was performed. Histopathological evaluation unexpectedly revealed the presence of a parasitic cyst consistent with cutaneous cysticercosis. The patient was clinically evaluated and found to have no further evidence of systemic disease. Cutaneous cysticercosis, although rare and often clinically silent, must be included in the differential diagnosis of subcutaneous swellings in endemic regions. This case emphasizes the role of imaging and histopathology in distinguishing parasitic infections from benign skin lesions and adds to the limited literature on isolated cutaneous forms.

## Introduction

Cutaneous cysticercosis is a localized manifestation of a systemic parasitic infection caused by *Taenia solium*, the pork tapeworm [1]. This condition arises due to hematogenous dissemination of the larval stage (cysticercus cellulosae) to subcutaneous tissues, muscles, or other organs [2]. Clinically, patients usually present with firm, mobile, cystic nodules that are often asymptomatic. These features commonly lead to misdiagnosis as benign dermatological conditions such as lipomas, epidermoid cysts, or fibromas [3].

In its subcutaneous form, cysticerci embed within subcutaneous or intramuscular tissues, presenting as painless, firm-to-soft nodules. Their asymptomatic nature and non-specific clinical appearance frequently result in diagnostic confusion with more common benign entities [4]. The subcutaneous form is particularly significant because it may represent the only clinical manifestation of cysticercosis in some patients. Its identification can serve as an important diagnostic clue for more serious manifestations, such as disseminated disease or neurocysticercosis [5]. Moreover, the presence of such lesions underscores ongoing zoonotic transmission and reflects broader public health challenges, especially in endemic regions.

Imaging plays a vital role in the evaluation of these lesions. Ultrasonography is a valuable, non-invasive, first-line tool for assessing superficial soft tissue abnormalities [6]. In cases of cysticercosis, ultrasound may reveal a well-defined cyst with an echogenic scolex, characteristically referred to as the “dot in cyst” sign. Nevertheless, in early stages or degenerated cysts, imaging findings can be non-specific and may mimic other benign conditions such as sebaceous or epidermoid cysts, as was observed in the present case [7]. Advanced imaging techniques such as CT or MRI are more appropriate for detecting deeper, muscular, or cerebral forms of the disease, particularly in cases where dissemination is suspected. However, they are not routinely employed for solitary, superficial lesions [8,9].

The life cycle of *T. solium* is complex, involving humans as definitive hosts and pigs as intermediate hosts. Accidental human infection occurs via ingestion of parasite eggs through feco-oral transmission. Once inside the gastrointestinal tract, the eggs hatch into larvae, penetrate the intestinal mucosa, and migrate via the bloodstream to lodge in various tissues, including the skin, muscle, brain, and eyes. Within these tissues, the cysticerci can evoke a range of immune responses. Viable cysts often generate minimal inflammation, whereas degenerating cysts provoke granulomatous reactions. Subcutaneous tissue, in particular, offers a relatively immune-privileged environment that permits long-term parasite survival with minimal host response [10].

Geographically, cysticercosis remains endemic in several parts of the world, including Latin America, sub-Saharan Africa, Southeast Asia, and the Indian subcontinent [11]. The disease burden is closely linked to poor sanitation, open defecation practices, and close contact between humans and pigs. Additionally, increased global migration has led to sporadic reports of cysticercosis in non-endemic areas, further highlighting the need for heightened clinical awareness across all healthcare settings [12].

## Case presentation

A 23-year-old male presented with a swelling over the right subcostal region, noticeable for the past two weeks. The swelling was not associated with pain or discharge. There was no history of trauma, fever, or systemic symptoms.

**Figure 1:**
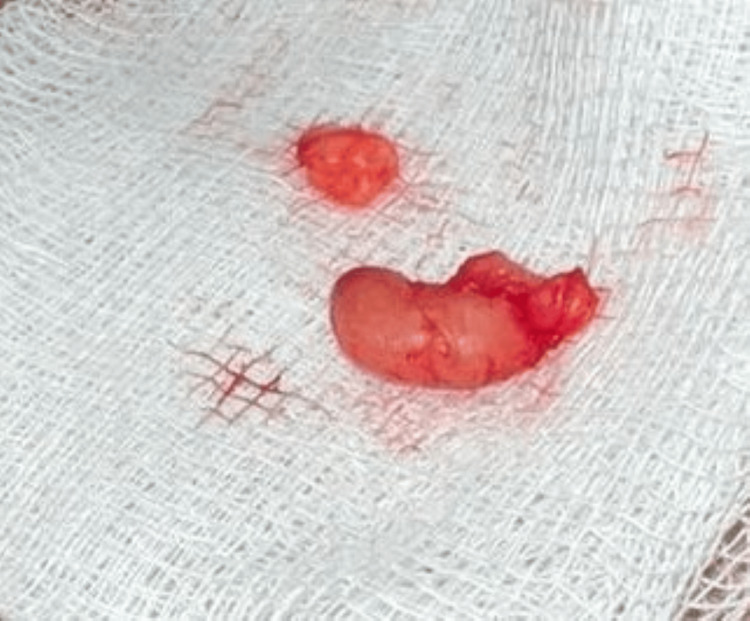
Gross excised specimen of cutaneous cysticercosis, a well-defined encapsulated lesion with cystic consistency.

On physical examination, a 0.5 × 1 cm, oval-shaped swelling was palpated. It exhibited a cystic consistency and was mobile and non-tender. No signs of inflammation or secondary changes were observed. High-frequency ultrasound of the superficial swelling revealed features suggestive of an epidermal inclusion cyst. Excisional biopsy of the lesion was performed for definitive diagnosis. Grossly, the excised specimen measured 0.5 × 1 cm, with a bean-shaped appearance and cystic consistency (Figure 1).

**Figure 2:**
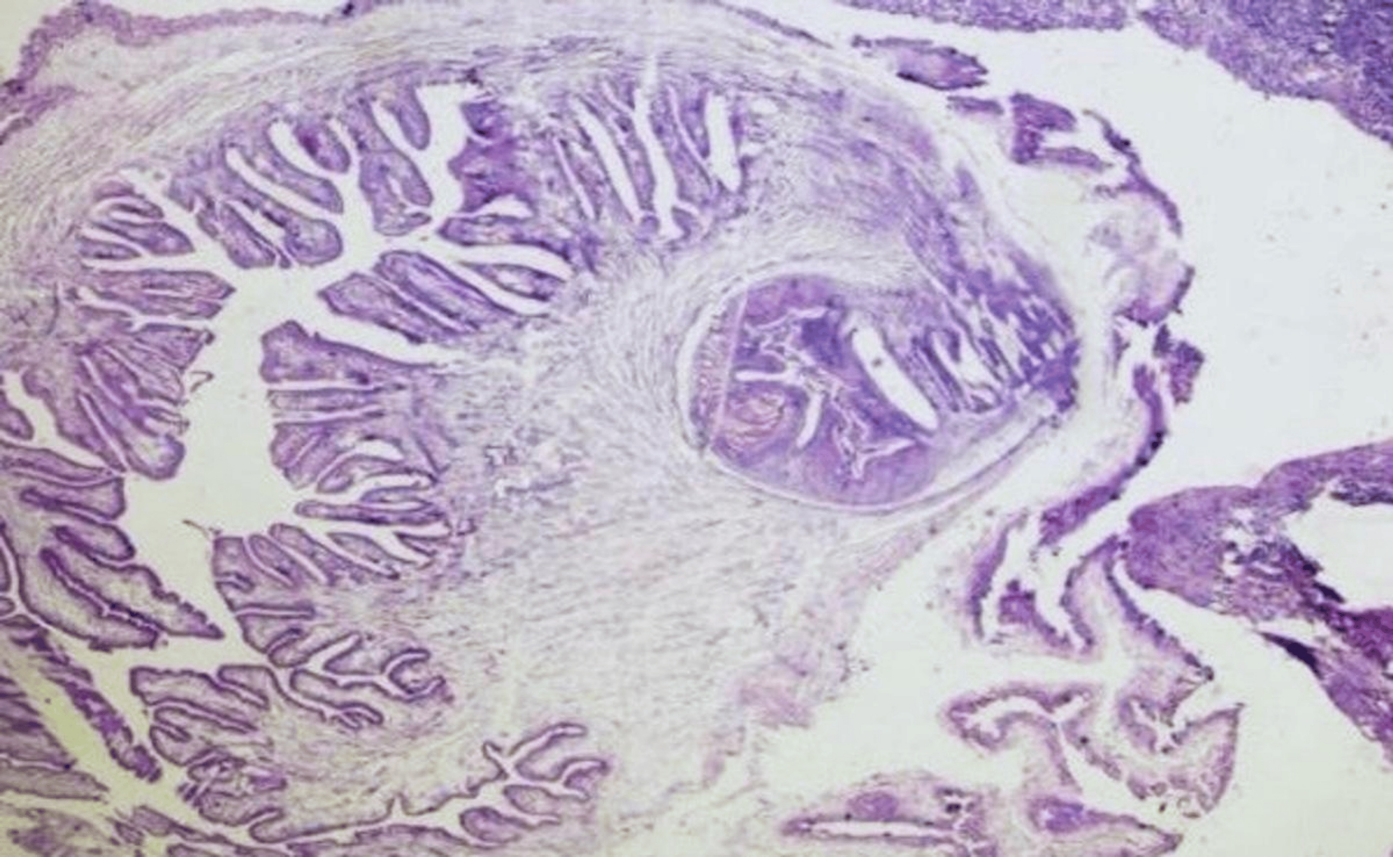
Histopathological examination showing irregularly shaped membranous foldings and scolices of Cysticercus larva, in a background of inflammatory infiltrate.

Surprisingly, histopathology did not confirm an epidermal inclusion cyst. Instead, it revealed features consistent with cutaneous cysticercosis, including irregularly shaped membranous foldings and *Cysticercus* larva with a background of chronic inflammatory infiltrate with eosinophilic preponderance and a granulomatous response (Figure 2).

The final diagnosis was confirmed as cutaneous cysticercosis masquerading as an epidermal inclusion cyst. On postoperative day five, the patient was reviewed. The incision site was healing well. In view of the histopathological examination report, a detailed clinical neurologic examination was performed, and no abnormality was detected. No other subcutaneous swelling was palpable anywhere on the body. As the patient was not willing to undergo further imaging and work-up, he was advised to take praziquantel 50 mg/kg/day for two weeks. The patient’s further recovery was uneventful.

## Discussion

In the current case, high-frequency ultrasonography demonstrated features suggestive of an epidermal inclusion cyst, a frequently encountered benign lesion characterized by a well-defined, hypoechoic, and avascular structure. However, definitive diagnosis was established on histopathological examination. This case is clinically significant due to its unusual presentation in the atypical subcostal site, as well as for its deceptive clinical presentation. The deceptive clinical and sonographic features underscore the diagnostic challenges associated with subcutaneous parasitic infestations, especially in regions where both benign cysts and parasitic infections are prevalent. It underscores the importance of including parasitic infections in the differential diagnosis of subcutaneous nodules, especially in endemic regions.

Cutaneous cysticercosis is typically acquired via hematogenous dissemination of *T. solium* larvae following ingestion of eggs through fecal-oral contamination. Although the cutaneous form is often asymptomatic and incidentally diagnosed, it may serve as an external marker for more severe systemic involvement, including neurocysticercosis. Hence, even in cases with solitary lesions, a thorough evaluation to rule out central nervous system involvement is essential. Neuroimaging, particularly MRI or CT brain scans, should be considered in patients exhibiting neurological symptoms or when multiple subcutaneous nodules are detected [13]. In this patient, the presence of a single lesion without neurological complaints or systemic involvement significantly reduced the likelihood of disseminated disease.

In this case, the absence of neurological symptoms or systemic signs, combined with the solitary lesion, made disseminated disease unlikely. Surgical excision served both diagnostic and therapeutic purposes. Current literature suggests that solitary, asymptomatic cutaneous cysticercosis may not necessitate antiparasitic therapy unless systemic involvement is evident. However, antihelminthic drugs remain a cornerstone in cases with systemic involvement.

Public health measures play a pivotal role in long-term prevention. Lifestyle choices, including proper sanitation and consumption of well-cooked meat, are essential in breaking the fecal-oral transmission cycle of *T. solium*. Ultimately, this case emphasizes the critical need for clinicians, especially in endemic areas, to maintain a broad and inclusive differential diagnosis when evaluating cutaneous swellings. Histopathological confirmation remains essential when there is diagnostic ambiguity. Prompt identification and management, whether surgical excision or medical therapy, can prevent complications and guide appropriate treatment strategies.

## Conclusions

Cutaneous cysticercosis, though often overlooked, is a clinically important yet under-recognized presentation of systemic *T. solium* infection. Its ability to mimic common benign skin lesions, such as epidermoid cysts or lipomas, frequently leads to misdiagnosis, particularly in regions where parasitic diseases are endemic. This case underscores the diagnostic value of histopathological examination in atypical cutaneous swellings and reinforces the role of high clinical suspicion, especially when imaging findings are inconclusive. Timely recognition of isolated cutaneous cysticercosis is crucial, not only for accurate management but also for evaluating the potential risk of disseminated or neurocysticercosis. Awareness among clinicians and integration of parasitic infections in differential diagnoses are essential steps toward effective disease surveillance and control in endemic and non-endemic areas alike.
